# The efficacy and safety of prasugrel in acute coronary syndrome: a propensity-matched Korean cohort study focused on age and weight of patients

**DOI:** 10.1186/s12872-025-05263-w

**Published:** 2025-11-26

**Authors:** Sang Hyun Kim, Eun Ho Choo, Jeehoon Kang, Jong-Min Lee, Ki-Dong Yoo, Mahn-Won Park, Chul Soo Park, Hee-Yeol Kim, Min Chul Kim, Youngkeun Ahn, Kiyuk Chang, Hyo-Soo Kim

**Affiliations:** 1 Division of Cardiology, Seoul St. Mary’s Hospital, The Catholic University of Korea, 222 Banpo-daero, Seocho-gu, Seoul, 06591 Republic of Korea; 2 Department of Internal Medicine, Division of Cardiology, The Armed Forces Capital Hospital, Seongnam, Korea; 3 Seoul National University Hospital, Seoul National University College of Medicine, Seoul, Korea; 4 Department of Critical Care Medicine, Seoul National University Hospital, Seoul, Republic of Korea; 5 Division of Cardiology, Uijeongbu St. Mary’s Hospital, The Catholic University of Korea, Uijeongbu, Korea; 6 Division of Cardiology, St. Vincent’s Hospital, The Catholic University of Korea, Suwon, Korea; 7 Division of Cardiology, Daejeon St. Mary’s Hospital, The Catholic University of Korea, Daejeon, Korea; 8 Division of Cardiology, Yeouido St. Mary’s Hospital, The Catholic University of Korea, Seoul, Korea; 9 Division of Cardiology, Bucheon St. Mary’s Hospital, The Catholic University of Korea, Bucheon, Korea; 10 Department of Cardiology, Chonnam National University Hospital, Gwangju, Korea

## Abstract

**Background:**

The clinical benefits of prasugrel compared to clopidogrel in acute coronary syndrome (ACS) patients undergoing percutaneous coronary intervention (PCI) are well established. However, significant safety concerns persist, particularly in elderly (≥ 75 years) or low-weight (< 60 kg) patients, even with reduced-dose prasugrel. We aimed to evaluate the comparative effectiveness and safety of prasugrel versus clopidogrel after PCI in Korean ACS patients, focusing on elderly or low-weight subgroup.

**Methods:**

We performed post-hoc analysis of data from the EFF-K and COREA-AMI II registries, including 3,744 prasugrel- and 9,588 clopidogrel-treated patients post-PCI. Propensity score (PS) matching created 3,725 patients per treatment arm to compare 1-year primary effectiveness (major adverse cardiac and cerebrovascular events [MACCE]: all-cause death, myocardial infarction, stroke, stent thrombosis, and any revascularization) and safety (Thrombolysis in Myocardial Infarction [TIMI] major or minor bleeding) outcomes. Subgroup analyses focused on age and weight of patients.

**Results:**

Among PS-matched patients, including 18.5% in the elderly (age ≥ 75 years) or low-body-weight (< 60 kg) (ELB) subgroup, the 1-year incidence of MACCE was significantly lower in the prasugrel arm (3.0% vs. 8.3%, HR 0.39, 95% CI 0.32–0.49, *P* < .001), with comparable TIMI bleeding rates (2.8% vs. 3.2%, HR 0.91, 95% CI 0.70–1.19, *P* = .495). In the ELB subgroup, 50.8% received 5 mg prasugrel. Prasugrel use was associated with a lower risk of MACCE (4.4% vs. 8.6%, HR 0.56, 95% CI 0.36–0.86, *P* = .009) without a significantly increased risk of bleeding (3.6% vs. 5.8%, HR 0.66, 95% CI 0.40–1.10, *P* = .111).

**Conclusions:**

In this Korean PS-matched ACS cohort, prasugrel, even at a reduced dose, was associated with superior effectiveness compared to clopidogrel without a significant increase in bleeding risk. These benefits were consistently observed in ELB patients. Our findings support the use of prasugrel in selected high-risk populations and offer guidance for clinical practice.

**Registration:**

EFF-K, Cris.nih.go.kr, Identifier: KCT0002356, registered June 13, 2017. COREA-AMI II, Clinicaltrials.gov, Identifier: NCT02806102, registered May 31, 2016

**Supplementary Information:**

The online version contains supplementary material available at 10.1186/s12872-025-05263-w.

## Introduction

Antiplatelet therapy is a cornerstone in long-term management of patients with acute coronary syndrome (ACS) following percutaneous coronary intervention (PCI) [1]. Recent major clinical studies have demonstrated the clinical benefits of a potent P2Y_12_ receptor inhibitor compared to clopidogrel, with prasugrel, in particular, being recommended over ticagrelor for ACS patients undergoing PCI [2–4]. However, stricter clinical indications for prasugrel are warranted due to safety concerns. The TRITON-TIMI 38 study demonstrated that two high-risk subgroups—patients aged 75 years or older and those weighing less than 60 kg—did not derive a net clinical benefit from prasugrel due to higher bleeding rates, despite a reduction in ischemic complications [5]. Consequently, current guidelines and drug labels advise against prasugrel use in these populations unless the anticipated benefit outweighs the bleeding risk, and recommend a reduced maintenance dose of 5 mg instead of the standard 10 mg [1, 2].

In clinical practice, prasugrel is widely administered to ACS patients at high ischemic risk; a group that includes elderly or low-weight individuals [6]. Moreover, older patients constitute a significant proportion of the ACS population, and increasing age is a well-established predictor of a poor prognosis [7]. Despite this, the evidence supporting the safety and efficacy of prasugrel in elderly or low-weight patients remains limited.

The aim of the present study is to assess the efficacy and safety of prasugrel in Korean patients with ACS undergoing PCI, and to further evaluate its impact in a high-risk subgroup of elderly (aged ≥ 75 years) or low-weight (< 60 kg) patients. By combining data from two large ACS registry cohorts from South Korea, we performed a comprehensive analysis comparing treatment with clopidogrel and prasugrel. Additionally, subgroup analyses were conducted to evaluate trends in elderly patients or low-weight patients.

## Methods

### Study design and patients

This study used combined data from two nationwide registries in South Korea: the EFF-K and COREA-AMI II (Cardiovascular Risk and Identification of Potential High-Risk Population in Acute Myocardial Infarction II) registries. Both are observational cohort studies that exclusively enrolled adult patients (≥ 19 years) diagnosed with ACS who underwent PCI during their index hospitalization. Patients managed conservatively without PCI were not included. In both registries, PCI was performed at the discretion of the treating physician in accordance with standard clinical guidelines.

The EFF-K registry prospectively enrolled patients who received prasugrel within six months after PCI at 52 hospitals from March 2017 to November 2019, while the COREA-AMI II registry enrolled consecutive acute myocardial infarction (MI) patients treated with PCI across nine major university cardiac centers from January 2010 to August 2014 [8]. As the COREA-AMI II registry preceded the widespread clinical uptake of prasugrel in Korea, clopidogrel remained the predominant P2Y_12_ inhibitor in this cohort. The EFF-K registry included two cohorts: a naïve cohort, in which prasugrel was administered as the initial P2Y_12_ inhibitor before PCI, and a switch cohort, in which patients were initially treated with another P2Y_12_ inhibitor (clopidogrel or ticagrelor) and later switched to prasugrel within six months after PCI. Reasons for switching included the need for greater antiplatelet potency, adherence consideration, or adverse event with prior agents, as described in prior publication [8]. Patients participating in other interventional studies involving antiplatelet or anticoagulant agents were excluded.

Data were collected prospectively in both registries, including demographic information, baseline clinical characteristics, procedural details, and clinical outcomes. In the EFF-K registry, clinical data collection began after enrollment (i.e. at the time the participant provided informed consent), particularly for patients who switched from another P2Y_12_ inhibitor. Follow-up data were obtained at predefined intervals up to one year after index PCI, with additional data recorded during unscheduled visits when applicable. All clinical outcomes were independently adjudicated by dedicated committees at each participating center, based on predefined criteria aligned with the universal definition of cardiovascular events [9], ensuring consistency across both registries. The study protocols for both registries were approved by the Institutional Review Boards of all participating institutions and complied with the Declaration of Helsinki. Written informed consent was obtained from all patients in the EFF-K registry, whereas it was waived by the Catholic Medical Center Institutional Review Board for those in the COREA-AMI II registry, due to minimal risk.

### Study outcomes and definitions

The primary efficacy outcome of the study was major adverse cardiac and cerebrovascular events (MACCE), a composite of all-cause death, MI, stroke, stent thrombosis, and any revascularization. The primary safety outcome was a composite of Thrombolysis in Myocardial Infarction (TIMI) major or minor bleeding events.

Subpopulations were defined as ELB (elderly patients aged ≥ 75 years or those with low body weight < 60 kg) and non-ELB (all other patients). Subgroup analyses were conducted to compare clinical outcomes between ELB and non-ELB patients, evaluating the impact of treatment (prasugrel vs. clopidogrel) on MACCE and bleeding events.

### Statistical analyses

Statistical analyses were performed using R version 4.3.1 (R Foundation for Statistical Computing, Vienna, Austria). Continuous variables are presented as mean ± standard deviation (SD) and were compared using independent sample t-tests or Mann-Whitney U-tests, as appropriate. Categorical variables are presented as frequency (percentage) and were compared using Pearson’s χ^2^ test or Fisher’s exact test, as appropriate.

Propensity score (PS) matching was performed to create balanced cohorts of prasugrel- and clopidogrel-treated patients based on relevant covariates. A PS analysis was performed to adjust potential confounders with a logistic regression model. Well-known risk factors for adverse clinical events were included as confounders: age, sex, body weight, hypertension, diabetes mellitus, chronic kidney disease, prior MI, previous bypass surgery, left ventricular ejection fraction (LVEF), diagnosis of ST elevation MI, culprit vessel, and multivessel disease. The predicted accuracy of the logistic model was assessed using the area under the receiver operating characteristic curve (C statistic), which was 0.697 (95% confidence intervals [CI] 0.687–0.707). Patients were matched 1:1 without replacement using the nearest neighbor method with a caliper width of 0.2 standard deviations (SD), a threshold shown to eliminate nearly 99% of bias in observed confounders. Baseline clinical and angiographic characteristics were compared within the PS-matched group, and balance between treatment groups was assessed using standardized mean differences (SMDs), with an SMD < 0.1 considered indicative of well-balanced covariates.

Kaplan-Meier survival curves were plotted to estimate the incidence of clinical outcomes within one year, and differences were compared using the log-rank test. Cox proportional hazards models were used to calculate hazard ratios (HRs) and 95% confidence intervals (Cis) for treatment effects. For the primary comparison between prasugrel and clopidogrel in the PS-matched cohorts, unadjusted Cox models were applied. In contrast, multivariable Cox models were constructed for the dose-stratified analysis limited to prasugrel-treated patients (5 mg vs. 10 mg), adjusting for carefully selected covariates to avoid overfitting. All tests were two-tailed, and a *P*-value < 0.05 was considered statistically significant.

## Results

### Patient selection and baseline characteristics

**Fig. 1:**
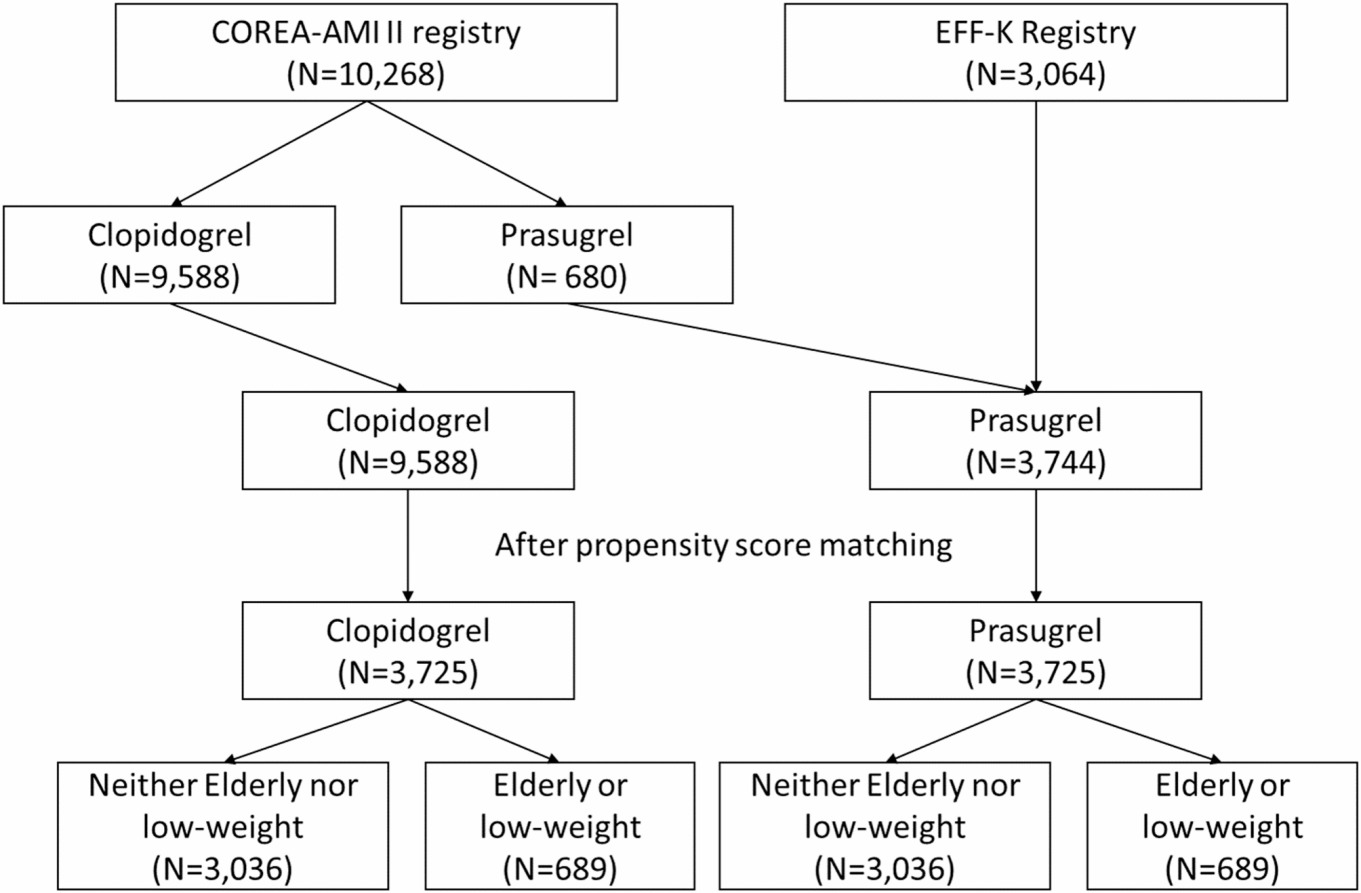
Patient selection process. Abbreviations: COREA-AMI II, Cardiovascular Risk and Identification of Potential High-Risk Population in Acute Myocardial Infarction II

The patient selection process is shown in Fig. 1. A total of 10,268 patients from the COREA-AMI II registry and 3,064 from the EFF-K registry were included. All patients in the EFF-K registry were treated with prasugrel, while the COREA-AMI II registry included 680 prasugrel-treated and 9,588 clopidogrel-treated patients. PS matching was performed using 9,588 clopidogrel- and 3,744 prasugrel-treated patients, resulting in 3,725 matched pairs.

In the prasugrel arm, 2,322 patients (62.3%) were enrolled from the switch cohort of the EFF-K registry, with a median [Q1, Q3] time from index PCI to prasugrel initiation of 1 [1, 13] day. The median [Q1, Q3] follow-up duration of the PS-matched cohort was 365 [348, 365] days. The ELB subgroup (age ≥ 75 years or low body weight < 60 kg) comprised 689 (18.5%) patients from each treatment arm.

Baseline characteristics of the overall PS-matched population and the ELB subgroup are presented in Table 1. In the ELB subgroup, patient characteristics were generally well-balanced between treatment arms. However, the prasugrel arm had a higher proportion of patients with dyslipidemia, non-smokers, a history of previous PCI, more vessels treated, and more frequent use of IVUS during index PCI. Among prasugrel-treated patients, 50.8% of the ELB subgroup received a reduced prasugrel dose of 5 mg, compared to 21.6% in the overall PS-matched cohort. Baseline characteristics of the total population before PS matching are shown in Additional file 1: Table S1.

**Table 1:** Baseline characteristics of PS matched study population

	Total cohort				ELB cohort			
	Prasugrel (*N* = 3725)	Clopidogrel (*N* = 3725)	*P* value	SMD	Prasugrel (*N* = 689)	Clopidogrel (*N* = 689)	*P* value	SMD
Clinical characteristics								
Age (yrs.)	59.8 ± 10.1	59.4 ± 11.0	0.162	0.032	68.8 ± 10.3	67.9 ± 11.0	0.122	0.083
≥ 75 years – no. (%)	283 (7.6)	254 (6.8)	0.210	0.030				0.086
Female Sex – no. (%)	588 (15.8)	675 (18.1)	0.008	0.062	323 (46.9)	329 (47.8)	0.787	0.017
Weight (kg)	70.2 ± 11.0	69.9 ± 10.8	0.145	0.034	57.2 ± 7.6	57.0 ± 8.4	0.728	0.019
< 60 kg – no. (%)	530 (14.2)	551 (14.8)	0.511	0.016				0.074
ELB – no. (%)	689 (18.5)	689 (18.5)	1.00	< 0.001				< 0.001
Prasugrel dose – no. (%)			NA				NA	
5 mg	806 (21.6)				350 (50.8)			
10 mg	2919 (78.4)				339 (49.2)			
Hypertension – no. (%)	1898 (51.0)	1875 (50.3)	0.610	0.012	409 (59.4)	399 (57.9)	0.623	0.029
Diabetes Mellitus - no. (%)	1090 (29.3)	1068 (28.7)	0.592	0.013	223 (32.4)	219 (31.8)	0.863	0.012
Dyslipidemia – no. (%)	1196 (32.1)	799 (21.4)	< 0.001	0.242	244 (35.4)	114 (16.5)	< 0.001	0.441
History of Smoking – no. (%)	1543 (41.4)	1712 (46.0)	< 0.001	0.092	143 (20.8)	177 (25.7)	0.035	0.117
Previous MI – no. (%)	159 (4.3)	151 (4.1)	0.685	0.011	34 (4.9)	28 (4.1)	0.516	0.042
Previous PCI – no. (%)	388 (10.4)	297 (8.0)	< 0.001	0.085	105 (15.2)	60 (8.7)	< 0.001	0.202
Previous CABG – no. (%)	10 (0.3)	20 (0.5)	0.100	0.042	3 (0.4)	4 (0.6)	1.00	0.020
Diagnosis – no. (%)			< 0.001	0.161			0.166	0.078
STEMI	1195 (32.1)	1481 (39.8)			178 (25.8)	202 (29.3)		
NSTEMI/UA	2530 (67.9)	2244 (60.2)			511 (74.2)	487 (70.7)		
eGFR ≤ 60 mL/min/1.73m^2^ – no. (%)	55 (1.5)	64 (1.7)	0.460	0.019	22 (3.2)	18 (2.6)	0.630	0.035
LVEF (%)	49.9 ± 20.6	54.7 ± 11.4	< 0.001	0.285	51.0 ± 19.4	53.4 ± 12.3	0.008	0.145
LVEF < 40% - no (%)	626 (16.8)	398 (10.7)	< 0.001	0.178	111 (16.1)	95 (13.8)	0.257	0.065
Angiographic characteristics								
Disease Extent – no (%)			0.759	0.017			0.233	0.092
1	1885 (50.6)	1875 (50.3)			333 (48.3)	321 (46.6)		
2	1145 (30.7)	1172 (31.5)			203 (29.5)	231 (33.5)		
3	695 (18.7)	678 (18.2)			153 (22.2)	137 (19.9)		
Culprit (%)			< 0.001	0.413			< 0.001	0.534
LM	164 (4.4)	121 (3.2)			31 (4.5)	28 (4.1)		
LAD	1688 (45.3)	1714 (46.0)			333 (48.3)	322 (46.7)		
LCX	561 (15.1)	730 (19.6)			84 (12.2)	137 (19.9)		
RCA	1026 (27.5)	1154 (31.0)			166 (24.1)	202 (29.3)		
Culprit of LM or LAD - no (%)	1852 (49.7)	1835 (49.3)	0.711	0.009	364 (52.8)	350 (50.8)	0.483	0.041
DES - no (%)	3393 (91.1)	3212 (86.2)	< 0.001	0.154	606 (88.0)	597 (86.6)	0.517	0.039
Number of Treated vessels	1.4 ± 0.7	1.3 ± 0.5	< 0.001	0.224	1.5 ± 0.7	1.3 ± 0.5	< 0.001	0.250
Total Stent length (mm)	24.0 ± 13.0	34.1 ± 21.2	< 0.001	0.576	22.9 ± 12.8	35.0 ± 21.6	< 0.001	0.681
IVUS - no (%)	1187 (31.9)	816 (21.9)	< 0.001	0.226	216 (31.3)	134 (19.4)	< 0.001	0.276

*Abbreviations*: *PS* Propensity score, *ELB* Elderly or low-body weighted, *MI* Myocardial infarction, *PCI* Percutaneous coronary intervention, *CABG* Coronary artery bypass grafting, (N)STEMI, (Non) ST-segment elevation myocardial infarction, *UA* Unstable angina, *LM* Left main coronary artery, *LAD* Left anterior descending artery, *LCX* Left circumflex artery, *RCA* Right coronary artery, *DES* Drug-eluting stent, *IVUS* Intravascular ultrasound, *LVEF* Left ventricular ejection fraction

### 1-year clinical outcomes in PS-matched patients

**Fig. 2:**
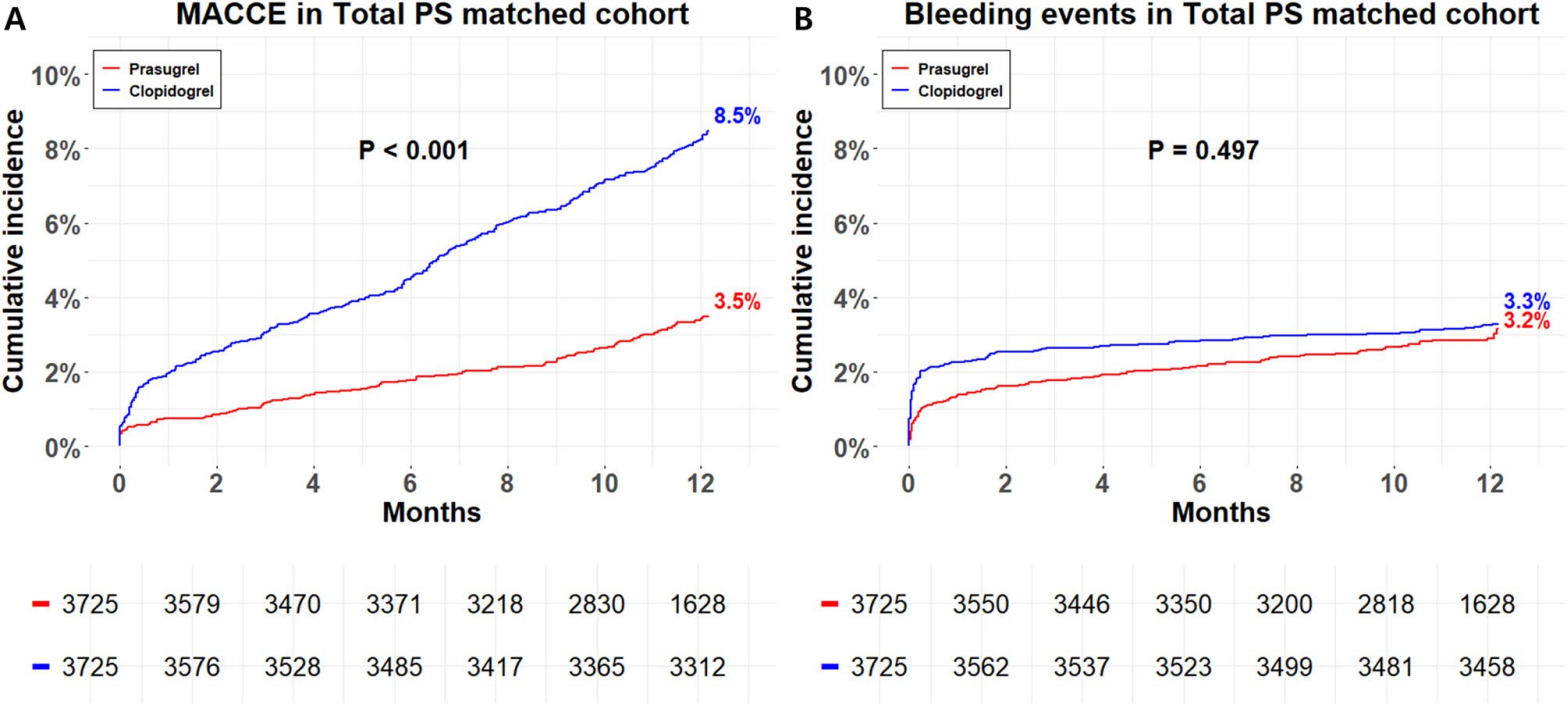
1-year clinical outcomes stratified by treatment arms among PS matched cohort. Kaplan–Meier curves for (A) MACCE and (B) TIMI major or minor bleeding events. Abbreviations: PS, Propensity score; MACCE, major adverse cardiac and cerebrovascular events; TIMI, Thrombolysis in Myocardial Infarction

Figure 2 illustrates the Kaplan-Meier curves for 1-year outcomes stratified by treatment arms in the overall PS-matched cohort. Prasugrel was associated with a significantly lower incidence of MACCE compared to clopidogrel (Prasugrel vs. Clopidogrel, 3.0% vs. 8.3%, HR 0.39, 95% CI 0.32–0.49, *P* <.001) (Table 2). The safety outcome (TIMI major or minor bleeding) was comparable between the treatment arms (2.8% vs. 3.2%, HR 0.91, 95% CI 0.70–1.19, *P* =.495).

**Table 2:** 1-year clinical outcomes according to treatment arms in PS matched cohort

	Total cohort				ELB cohort			
	Prasugrel (*n* = 3725)	Clopidogrel (*n* = 3725)	HR (95% CI)	*P* value	Prasugrel (*n* = 689)	Clopidogrel (*n* = 689)	HR (95% CI)	*P* value
Primary ischemic outcome								
MACCE (Composite of all-cause death, MI, stroke, stent thrombosis and any revascularization)	112 (3.0)	310 (8.3)	0.39 (0.32–0.49)	< 0.001	30 (4.4)	59 (8.6)	0.56 (0.36–0.86)	0.009
All cause of death	24 (0.6)	42 (1.1)	0.63 (0.38–1.04)	0.071	11 (1.6)	17 (2.5)	0.71 (0.33–1.51)	0.371
Cardiovascular cause death	8 (0.2)	39 (1.0)	0.22 (0.10–0.47)	< 0.001	3 (0.4)	14 (2.0)	0.22 (0.06–0.78)	0.018
MI	30 (0.8)	65 (1.7)	0.50 (0.32–0.76)	0.001	7 (1.0)	14 (2.0)	0.56 (0.23–1.39)	0.212
Stent thrombosis	25 (0.7)	25 (0.7)	1.06 (0.61–1.84)	0.845	6 (0.9)	8 (1.2)	0.79 (0.27–2.27)	0.658
Revascularization	40 (1.1)	222 (6.0)	0.20 (0.14–0.28)	< 0.001	11 (1.6)	38 (5.5)	0.33 (0.17–0.65)	0.001
Stroke	14 (0.4)	42 (1.1)	0.35 (0.19–0.64)	< 0.001	3 (0.4)	11 (1.6)	0.28 (0.08–1.01)	0.052
Primary safety outcome								
TIMI major or minor bleeding	105 (2.8)	121 (3.2)	0.91 (0.70–1.19)	0.495	25 (3.6)	40 (5.8)	0.66 (0.40–1.10)	0.111
Net adverse clinical outcomes								
Composite of MACCE, TIMI major or minor bleeding	206 (5.5)	406 (10.9)	0.54 (0.46–0.64)	< 0.001	51 (7.4)	90 (13.1)	0.60 (0.43–0.85)	0.004

*Abbreviations*: *PS* Propensity score, *ELB* Elderly or Low-body weight, *HR* hazard ratio, *CI* confidence interval, *MACCE* major adverse cardiac and cerebrovascular events, *MI* myocardial infarction, *TIMI* Thrombolysis in Myocardial Infarction

### 1-year clinical outcomes in the ELB subgroup

**Fig. 3:**
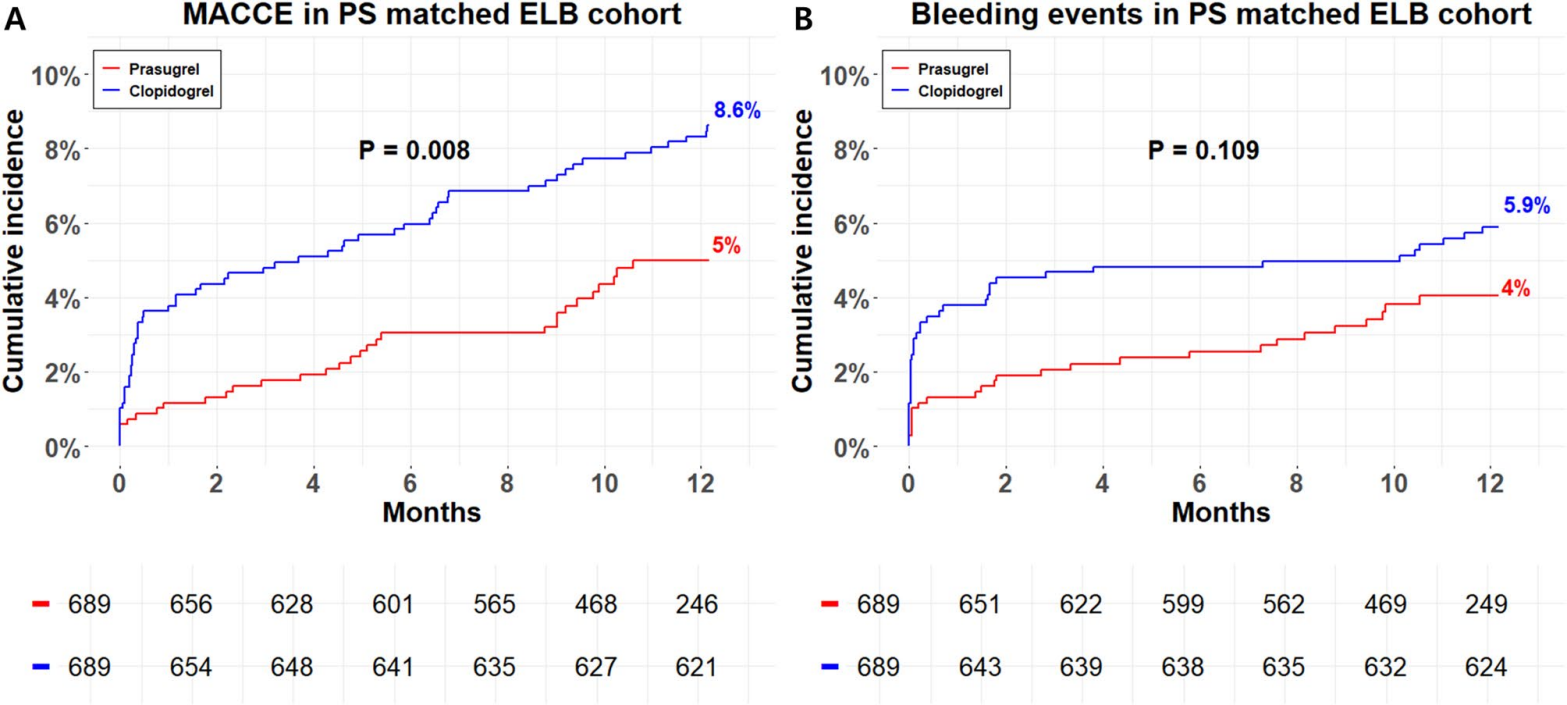
1-year clinical outcomes stratified by treatment arms among PS matched ELB cohort. Kaplan–Meier curves for (**A**) MACCE and (**B**) TIMI major or minor bleeding events. Abbreviations: PS, Propensity score; ELB, Elderly or Low-body weight; MACCE, major adverse cardiac and cerebrovascular events; TIMI, Thrombolysis in Myocardial Infarction

Figure 3 demonstrates the clinical outcomes in the ELB subgroup. Prasugrel was associated with a significantly lower incidence of 1-year MACCE compared to clopidogrel (4.4% vs. 8.6%, HR 0.56, 95% CI 0.36–0.86, *P* =.009), without a significant increase in TIMI bleeding (3.6% vs. 5.8%, HR 0.66, 95% CI 0.40–1.10, *P* =.111). Kaplan-Meier curves for the non-ELB cohort are shown in Additional file 1: Fig. S1.

### Interaction analysis and subgroup evaluation

**Fig. 4:**
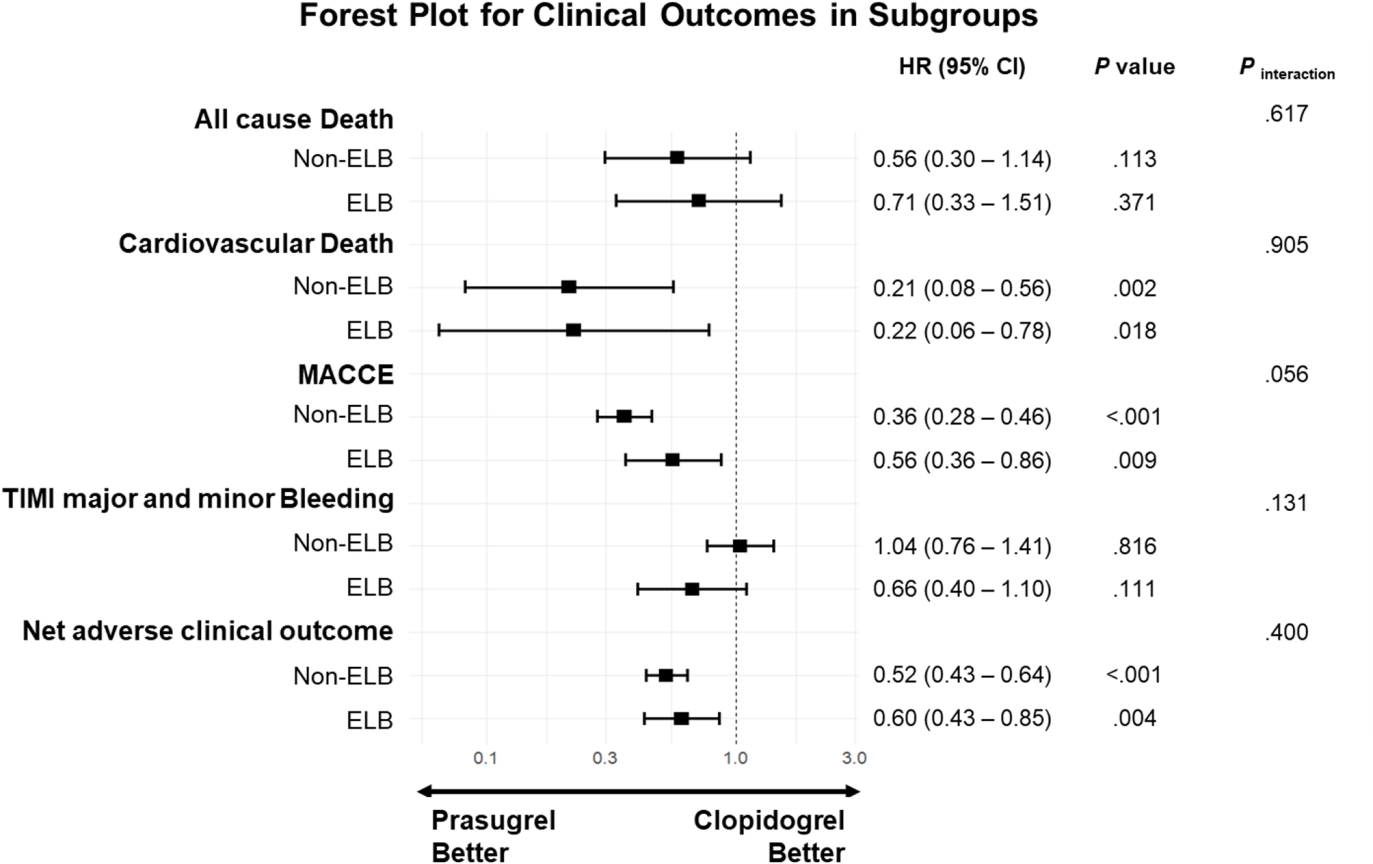
Associations of the antiplatelet strategy and 1-year clinical outcomes in PS matched subgroups. Abbreviations: PS, Propensity score; ELB, Elderly or low-body weighted; HR, Hazard Ratio; CI, Confidential interval; MACCE, major adverse cardiac and cerebrovascular events; TIMI, Thrombolysis in Myocardial Infarction

The association between antiplatelet strategy (prasugrel vs. clopidogrel) and 1-year clinical outcomes in PS-matched ELB and non-ELB patients is shown in Fig. 4. No significant interaction was observed between the treatment arm and ELB status regarding clinical outcomes, including the primary efficacy outcome (*P*_interaction_ = 0.056) and the safety outcome (*P*_interaction_ = 0.131). The net adverse clinical outcome, defined as a composite of primary efficacy and safety outcomes, was significantly lower in both the ELB and non-ELB subgroups (*P*_interaction_ = 0.400). The results of Cox proportional hazard model evaluating the treatment effect on clinical outcomes in the ELB and non-ELB cohorts are detailed in Additional file 1: Table S2. Subgroup analyses for MACCE and bleeding events across key baseline variables are presented in Additional file 1: Fig. S2.

### Dose-stratified analysis within prasugrel-treated patients

A dose-stratified analysis (5 mg vs. 10 mg prasugrel) was conducted within ELB and non-ELB subgroups, and the results are presented in Additional file 1: Table S3. In the non-ELB subgroup, 10 mg prasugrel was associated with a significantly higher risk of net adverse clinical events, although individual MACCE and bleeding risks were not significantly different. In the ELB subgroup, no statistically significant differences in outcomes were observed between dose groups. No interaction by ELB status was detected for dose effect.

## Discussion

Our real-world, propensity-matched Korean cohort study, consisting exclusively of ACS patients undergoing PCI, included a substantial proportion of elderly (≥ 75 years) or low-weight (< 60 kg) individuals. Nearly half of the PS-matched elderly or low-weight cohort received a reduced 5 mg dose of prasugrel. In the overall PS-matched cohort, prasugrel was associated with a significantly lower incidence of 1-year MACCE compared to clopidogrel, without a significant increase in bleeding events. These favorable safety and efficacy trends were similarly observed in the elderly or low-weight subgroup, with no significant interaction effects detected.

The primary strength of this analysis lies in its large sample size and real-world applicability, encompassing a nationwide cohort of ACS patients in Korea. While the ISAR-REACT5 substudy demonstrated comparable efficacy of reduced-dose prasugrel with the standard dose of ticagrelor in elderly or low-weight patients, few studies have focused specifically on this high-risk ACS population [10]. Notably, clinical trials in East Asian populations, where bleeding risks are heightened (the “East Asian paradox”), often excluded elderly or frail patients or those receiving reduced-dose prasugrel [11, 12]. In this context, real-world data reflecting clinical practice with the reduced 5 mg dose of prasugrel in East Asians remain scarce. Our study provides unique insights into prasugrel use in underrepresented high-risk groups, including those treated with the 5 mg dose [13].

Invasive strategies remain central to the treatment of elderly ACS patients, with evidence suggesting comparable or better outcomes than conservative approaches [14–16]. Unlike the general population, questions persist regarding the benefit of a potent P2Y_12_ inhibitor, particularly prasugrel, in managing elderly or low-weight patients post-PCI [17]. However, the clinical demand for prasugrel remains valid in this ACS population due to its potent anti-ischemic benefits, driven by angiographic or clinical properties, and its advantage of once-daily dosing for improved medication compliance [8, 18]. Despite these clinical needs, the heightened bleeding risk associated with prasugrel, especially in elderly or frail populations, remains a major concern, even with a reduced dose [11, 19]. Although the relatively small portion of elderly or low-weight ACS patients warrants further investigation, current analyses from Korean registries align with prior studies supporting the anti-ischemic benefit with adequate safety of prasugrel in this subpopulation [10]. Considering the high bleeding risk in this ethnicity or age group, the comparable bleeding rates observed alongside a significant reduction in ischemic events highlight prasugrel’s potential to improve clinical outcomes in these high-risk populations.

While our study focused on real-world prasugrel use in East Asian patients with ACS, ethical concerns remain regarding its use in individuals at high risk of bleeding. In a nationwide Japanese study by Shoji et al., a 3.75 mg/day dose of prasugrel (an approved regimen in Japan) was associated with a higher bleeding risk than clopidogrel [20]. This elevated bleeding risk was particularly evident among patients with high bleeding risk profiles, such as those aged ≥ 75 years or weighing less than 60 kg. These findings underscore the importance of careful bleeding risk assessment, even when using regionally approved lower-dose regimens, particularly in older or low-body-weight patients [21]. In our cohort, prasugrel dosing was guided by physician judgement based on individualized evaluation of ischemic and bleeding risks. The observed outcomes, which include a substantial proportion of patients treated with the 5 mg dose, reflect real-world rather than protocol-driven practice.

The clinical applicability of reduced-dose prasugrel in elderly or low-weight patients has been evaluated across various settings. In the TRITON-TIMI 38 trial, the elevated bleeding risk offset its ischemic benefit in these subgroups [2], though subsequent studies supported the 5 mg dose for overcoming high platelet reactivity in clopidogrel-treated patients [22–24]. However, clinical outcomes from reduced-dose prasugrel vary by the study design. In a medically managed elderly ACS cohort without ST-segment elevation, 5 mg prasugrel was not associated with improved ischemic outcomes or increased bleeding events compared to clopidogrel [25]. Similarly, the Elderly ACS2 reported comparable 12-month ischemic outcomes between reduced-dose prasugrel and clopidogrel in elderly PCI-treated ACS patients, with numerically fewer stent thrombosis but a higher bleeding rate in the prasugrel group [6]. In Japanese studies evaluating 3.75 mg prasugrel, the PRASFIT-ACS trial demonstrated a favorable safety and efficacy profile in an ACS population including elderly or low-weight patients [26], while a registry-based study reported increased in-hospital bleeding events compared to clopidogrel, despite similar in-hospital mortality and stent thrombosis rates [27].

Increasing age is an independent risk factor for poor clinical outcomes in ACS, and older, underweight, frail populations often exhibit high ischemic features, multiple comorbidities, and an elevated bleeding risk [28]. Recent clinical studies have demonstrated that novel strategies, such as adjusting the duration or potency of antiplatelet agents following PCI, can significantly reduce bleeding complications, while maximizing the prevention of ischemic events during the high-ischemic period after ACS [29, 30]. Our findings, based on the clinically available 5 mg or 10 mg dose of prasugrel, support its use as a viable treatment option for East Asian patients with ACS undergoing PCI, showing consistent efficacy and safety in elderly or low-weight patients for whom a reduced dose is indicated. Clinicians should consider the cautious use of prasugrel in the elderly or low-weight population, carefully balancing the benefits of reduced ischemic events against the potential risk of bleeding complications.

Although the treatment arms showed differences in the incidence of MACCE, it is important to note the overall low event rates of 3.0% in the prasugrel arm. This is notably lower than rates reported in prior randomized trials such as TRITON-TIMI 38 or ISAR-REACT 5, despite our primary composite outcome including all-cause death, MI, stroke, and ischemia-driven revascularization—a broader definition of ischemic events [2, 4]. Several factors may explain this discrepancy. First, our study population was drawn from more contemporary registries, particularly the EFF-K registry (2017–2019), reflecting recent advancements in PCI techniques, stent platforms, and medical therapy, all of which may have contributed to improved outcomes. Second, the EFF-K registry included a substantial proportion of patients from the switch cohort, in whom prasugrel was initiated after the index PCI, and clinical events were collected from the time of prasugrel initiation. As a result, early post-PCI events, including periprocedural complications, may have been missed, potentially underestimating the true incidence of ischemic events in the prasugrel arms. Third, although the follow-up duration was comparable to those in prior trials, the clinical events captured through combined, registry-based data may differ from those collected in randomized trials with uniform, centrally adjudicated outcome reporting. Lastly, our study population was predominantly East Asian, and this group is known to have lower baseline ischemic risk, which is often referred to as the East Asian paradox [31]. Moreover, although the pharmacologic data were not available in this study, East Asian populations have a high prevalence of CYP2C19 loss-of-function alleles, which may reduce clopidogrel responsiveness and enhance the relative ischemic benefit of prasugrel [32, 33]. Taken together, these factors likely account for the relatively low ischemic event rate observed in the prasugrel group.

This study has several limitations. First, although propensity score matching was employed to balance key baseline characteristics, some residual imbalances—such as dyslipidemia and previous history of PCI—persisted between groups. The non-randomized, observational nature of the current analysis inherently limits control over unmeasured confounders, including adherence to medication, frailty or socioeconomic status, which may be particularly relevant in elderly or low-weight populations. Therefore, while the statistical approach aimed to improve comparability, the findings should be interpreted as hypothesis-generating rather than establishing causality. Second, due to study design of the EFF-K registry, which allowed inclusion of patients who have switched to prasugrel from another P2Y_12_ inhibitor up to six months post-PCI, early clinical outcomes including in-hospital events may have been underestimated. Although this design enabled inclusion of a larger real-world cohort, it inherently excluded acute-phase outcomes for a substantial subset of patients, potentially biasing comparison with the clopidogrel group. Third, the prasugrel dosing was not randomized but determined at the discretion of treating physician, which may have introduced treatment selection bias. Nonetheless, the stratified analysis showed similar patterns of clinical outcomes between ELB and non-ELB groups, and no statistically significant interaction was observed, suggesting no effect modification by ELB status. Fourth, the study period of COREA-AMI II precedes that of EFF-K by less than a decade, but advancements in interventional or medical management during this interval may have contributed to more favorable clinical outcome in the prasugrel group. Fifth, the genetic data related to CYP2C19 metabolizer status were not available in either registry, limiting our ability to account for pharmacogenetic differences. This is particularly relevant in the East Asian population, where the prevalence of CYP2C19 loss-of-function alleles is high and associated with reduced clopidogrel effectiveness [34]. The absence of genotype data may have influenced comparative outcomes, potentially exaggerating the apparent benefit of prasugrel in this population. Future studies incorporating pharmacogenetic profiling are warranted to refine antiplatelet strategy selection. Finally, although the study included a large number of patients, it was conducted in South Korea with a predominantly East Asian population, which may restrict the generalizability of the findings to other ethnicities or regions.

## Conclusion

Our analysis on real-world Korean registries provides evidence supporting the use of prasugrel, even at a reduced dose, in elderly or low-weight patients with ACS undergoing PCI. These findings highlight prasugrel’s potential for safe and effective use in high-risk populations, offering valuable insights for clinical practice and guideline recommendations.

## Supplementary Information

Supplementary Material 1

## Data Availability

The datasets generated and/or analyzed during the current study are available from the corresponding author on reasonable request.
