# Supplementary material for: The efficacy and safety of prasugrel in acute coronary syndrome: a propensity-matched Korean cohort study focused on age and weight of patients
Source: BMC Cardiovasc Disord. 2025 Nov 26;25:888. doi: 10.1186/s12872-025-05263-w (PMC12751691; doi:10.1186/s12872-025-05263-w)
Supplement: Supplementary file 1 — Supplementary Material 1 [file 12872_2025_5263_MOESM1_ESM.docx]

**Table S1**. Baseline characteristics of study population before PS matching.

|  | **Prasugrel**  **(N=3744)** | **Clopidogrel**  **(N=9588)** | ***P* value** |
| --- | --- | --- | --- |
| Age-yrs. | 59.8 ± 10.2 | 64.2 ± 12.8 | <.001 |
| Female Sex (%) | 593 (15.8) | 2866 (29.9) | <.001 |
| Weight (kg) | 70.2 ± 11.0 | 64.6 ± 11.9 | <.001 |
| ELB – no. (%) | 694 (18.5) | 3906 (40.7) | <.001 |
| Prasugrel dose – no. (%) |  |  | NA |
| 5mg | 811 (21.7) |  |  |
| 10mg | 2933 (78.3) |  |  |
| Hypertension – no. (%) | 1907 (50.9) | 5115 (53.3) | .013 |
| Diabetes Mellitus - no. (%) | 1098 (29.3) | 3053 (31.8) | .005 |
| Dyslipidemia – no. (%) | 1201 (32.1) | 1443 (15.1) | <.001 |
| History of Smoking – no. (%) | 1832 (59.8) | 0 (0.0) | <.001 |
| eGFR ≤ 60 -no. (%) | 55 (1.5) | 222 (2.3) | .003 |
| Previous MI – no. (%) | 159 (4.2) | 410 (4.3) | .978 |
| Previous PCI – no. (%) | 390 (10.4) | 688 (7.2) | <.001 |
| Previous CABG – no. (%) | 10 (0.3) | 50 (0.5) | .068 |
| Diagnosis – no. (%) |  |  | <.001 |
| STEMI | 1202 (32.1) | 5174 (54.0) |  |
| NSTEMI/UA | 2542 (67.9) | 4414 (46.0) |  |
| Disease Extent – no (%) |  |  | <.001 |
| 1 | 1892 (50.5) | 4342 (45.3) |  |
| 2 | 1153 (30.8) | 3097 (32.3) |  |
| 3 | 699 (18.7) | 2149 (22.4) |  |
| Culprit (%) |  |  | <.001 |
| LAD | 1696 (45.3) | 4588 (47.9) |  |
| LCX | 568 (15.2) | 1579 (16.5) |  |
| LM | 166 (4.4) | 312 (3.3) |  |
| RCA | 1028 (27.5) | 3088 (32.2) |  |
| DES - no (%) | 3411 (91.1) | 8310 (86.7) | <.001 |
| Number of Treated vessels | 1.4 ± 0.7 | 1.3 ± 0.5 | <.001 |
| Total Stent length (mm) | 24.0 ± 13.0 | 34.1 ± 20.7 | <.001 |
| IVUS - no (%) | 1191 (31.8) | 1976 (20.6) | <.001 |
| LVEF (%) | 49.9 ± 20.6 | 52.9 ± 11.5 | <.001 |

* Abbreviations: PS, Propensity score; ELB, Elderly or low-body weighted; MI, Myocardial infarction; PCI, Percutaneous coronary intervention; CABG, Coronary artery bypass grafting; (N)STEMI, (Non) ST-segment elevation myocardial infarction; UA, Unstable angina; LM, Left main coronary artery; LAD, Left anterior descending artery; LCX, Left circumflex artery; RCA, Right coronary artery; DES, Drug-eluting stent; IVUS, Intravascular ultrasound; LVEF, Left ventricular ejection fraction.

**Table S2. 1-year clinical outcomes according to treatment arms in PS matched ELB and non-ELB cohort**

|  | **Non-ELB cohort** | | | | **ELB cohort** | | | |
| --- | --- | --- | --- | --- | --- | --- | --- | --- |
|  | **Clopidogrel**  **(n = 3036)** | **Prasugrel**  **(n = 3036)** | **HR (95% CI)** | ***P* value** | **Clopidogrel**  **(n = 689)** | **Prasugrel**  **(n = 689)** | **HR (95% CI)** | ***P* value** |
| Primary ischemic outcome |  |  |  |  |  |  |  |  |
| MACCE (Composite of all-cause death, MI, stroke, stent thrombosis and any revascularization) | 251 (8.3) | 82 (2.7) | 0.36 (0.28 - 0.46) | <.001 | 59 (8.6) | 30 (4.4) | 0.56 (0.36 - 0.86) | .009 |
| All cause of death | 25 (0.8) | 13 (0.4) | 0.58 (0.30 - 1.14) | .113 | 17 (2.5) | 11 (1.6) | 0.71 (0.33 - 1.51) | .371 |
| Cardiovascular cause death | 25 (0.8) | 5 (0.2) | 0.21 (0.08 - 0.56) | .002 | 14 (2.0) | 3 (0.4) | 0.22 (0.06 - 0.78) | .018 |
| MI | 51 (1.7) | 23 (0.8) | 0.48 (0.29 - 0.79) | .004 | 14 (2.0) | 7 (1.0) | 0.56 (0.23 - 1.39) | .212 |
| Stent thrombosis | 17 (0.6) | 19 (0.6) | 1.19 (0.62 - 2.29) | .610 | 8 (1.2) | 6 (0.9) | 0.79 (0.27 - 2.27) | .658 |
| Revascularization | 184 (6.1) | 29 (1.0) | 0.18 (0.12 - 0.26) | <.001 | 38 (5.5) | 11 (1.6) | 0.33 (0.17 - 0.65) | .001 |
| Stroke | 31 (1.0) | 11 (0.4) | 0.37 (0.19 - 0.74) | .005 | 11 (1.6) | 3 (0.4) | 0.28 (0.08 - 1.01) | .052 |
| Primary safety outcome |  |  |  |  |  |  |  |  |
| TIMI major or minor bleeding | 81 (2.7) | 80 (2.6) | 1.04 (0.76 - 1.41) | .816 | 40 (5.8) | 25 (3.6) | 0.66 (0.40 - 1.10) | .111 |
| Net adverse clinical outcomes |  |  |  |  |  |  |  |  |
| Composite of MACCE, TIMI major or minor bleeding | 316 (10.4) | 155 (5.1) | 0.52 (0.43 - 0.64) | <.001 | 90 (13.1) | 51 (7.4) | 0.60 (0.43 - 0.85) | .004 |

Abbreviations: PS, Propensity score; ELB, Elderly or low-body weighted; HR, hazard ratio; CI, confidence interval; MACCE, major adverse cardiac and cerebrovascular events; MI, myocardial infarction; TIMI, Thrombolysis in Myocardial Infarction.

**Table S3. 1-year clinical outcomes according to prasugrel dose in in PS matched cohort**

|  | **Non-ELB cohort** | | | | **ELB cohort** | | | |  |
| --- | --- | --- | --- | --- | --- | --- | --- | --- | --- |
|  | **Prasugrel**  **5 mg**  **(n = 456)** | **Prasugrel**  **10 mg**  **(n = 2580)** | ***Adjusted HR (95% CI)** | ***P* value** | **Prasugrel**  **5 mg**  **(n = 350)** | **Prasugrel**  **10 mg**  **(n = 339)** | ***Adjusted HR (95% CI)** | ***P* value** | ***P* _interaction_** |
| Primary ischemic outcome |  |  |  |  |  |  |  |  |  |
| MACCE (Composite of all-cause death, MI, stroke, stent thrombosis and any revascularization) | 7 (1.5) | 75 (2.9) | 1.50 (0.68 - 3.32) | .313 | 10 (2.9) | 20 (5.9) | 1.83 (0.76 - 4.44) | .179 | .856 |
| All cause of death | 1 (0.2) | 12 (0.5) | 1.57 (0.19 - 12.6) | .673 | 7 (2.0) | 4 (1.2) | 0.45 (0.08 - 2.39) | .348 | .288 |
| Cardiovascular cause death | 0 (0.0) | 5 (0.2) | NA | .999 | 1 (0.3) | 2 (0.6) | NA | .841 | .998 |
| MI | 2 (0.4) | 21 (0.8) | 1.37 (0.31 - 6.03) | .673 | 2 (0.6) | 5 (1.5) | 1.74 (0.30 - 10.1) | .536 | .771 |
| Stent thrombosis | 2 (0.4) | 17 (0.7) | 1.28 (0.28 - 5.81) | .745 | 0 (0.0) | 6 (1.8) | NA | .999 | .996 |
| Revascularization | 2 (0.4) | 27 (1.0) | 1.66 (0.38 - 7.19) | .499 | 1 (0.3) | 10 (2.9) | 7.99 (0.88 - 72.9) | .065 | .248 |
| Stroke | 1 (0.2) | 10 (0.4) | 1.81 (0.22 - 14.6) | .579 | 1 (0.3) | 2 (0.6) | 4.78 (0.12 - 183.9) | .401 | .926 |
| Primary safety outcome |  |  |  |  |  |  |  |  |  |
| TIMI major or minor bleeding | 5 (1.1) | 75 (2.9) | 2.34 (0.93 - 5.88) | .069 | 11 (3.1) | 14 (4.1) | 1.57 (0.63 - 3.92) | .338 | .252 |
| Net adverse clinical outcomes |  |  |  |  |  |  |  |  |  |
| Composite of MACCE, TIMI major or minor bleeding | 11 (2.4) | 144 (5.6) | 1.99 (1.07 - 3.71) | .031 | 19 (5.4) | 32 (9.4) | 1.72 (0.89 - 3.33) | .105 | .527 |

*Multivariable models adjusted for: Age ≥75, female sex, body weight < 60kg, hypertension, diabetes mellitus, smoking status, dyslipidemia, eGFR < 60, prior history of PCI, clinical presentation, LAD culprit vessel, stent length, and LVEF < 40%.

Abbreviations: PS, Propensity score; HR, hazard ratio; CI, confidence interval; MACCE, major adverse cardiac and cerebrovascular events; MI, myocardial infarction; TIMI, Thrombolysis in Myocardial Infarction, eGFR, estimated glomerulus filtration rate, PCI, percutaneous coronary intervention, LAD, left anterior descending coronary artery, DES, drug eluting stent, LVEF, left ventricular ejection fraction.

**Figure S1.** 1-year clinical outcomes stratified by treatment arm among PS matched non-ELB patient


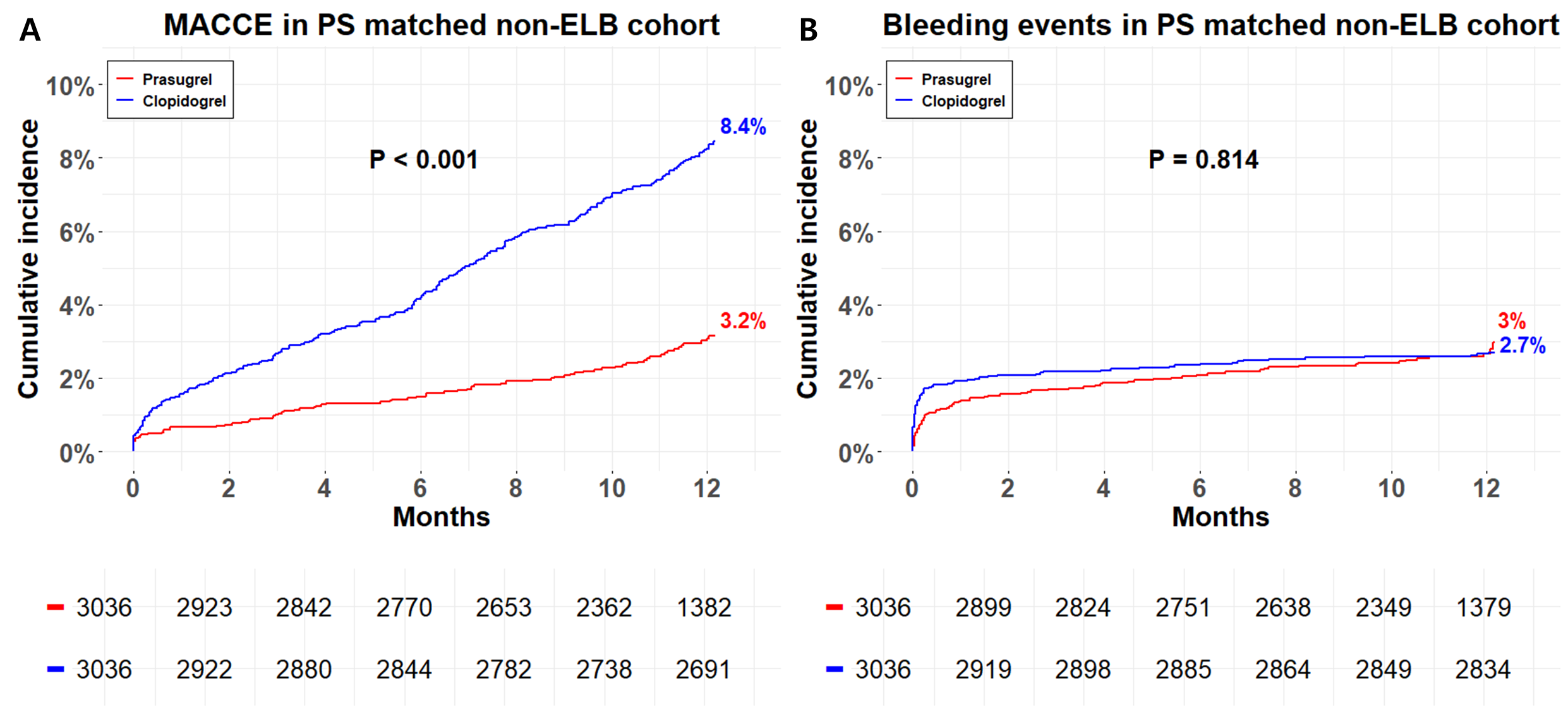


Kaplan–Meier curves for (A) MACCE and (B) TIMI major or minor bleeding events.

* Abbreviations: PS, Propensity score; ELB, Elderly or lower-body weighted; MACCE, major adverse cardiac and cerebrovascular events; TIMI, Thrombolysis in Myocardial Infarction.

**Figure S2.** Subgroup analyses comparing antiplatelet strategy on ischemic or bleeding events in the PS matched ELB or non-ELB cohorts


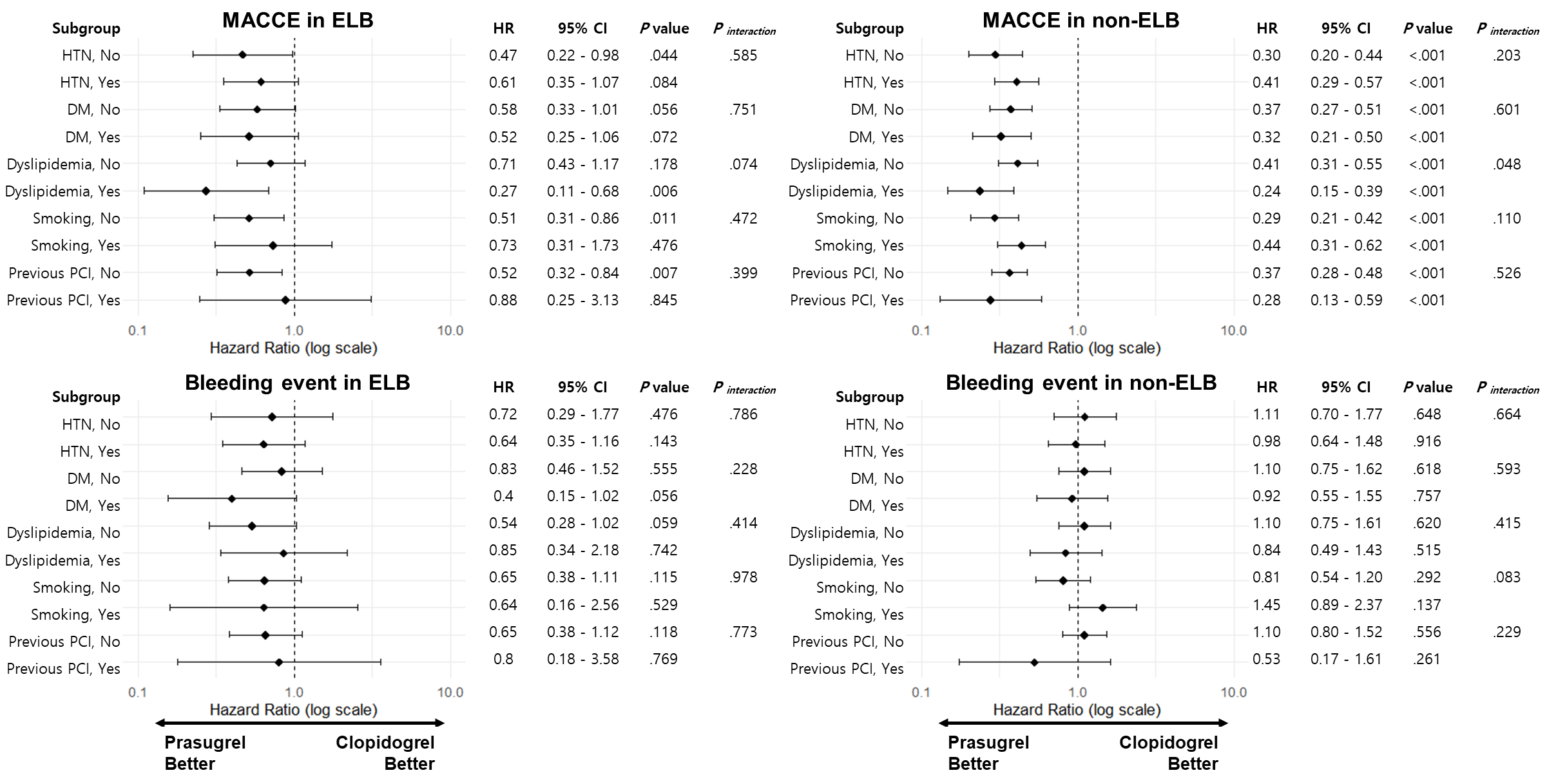


* Abbreviations: PS, Propensity score; ELB, Elderly or low-body weighted; HR, Hazard Ratio; CI, Confidential interval; MACCE, major adverse cardiac and cerebrovascular events; HTN, Hypertension; DM, diabetes mellitus; PCI, percutaneous coronary intervention.
